# Impact of somatic mutations on response to lenalidomide in lower-risk non-del(5q) myelodysplastic syndromes patients

**DOI:** 10.1038/s41375-020-0961-3

**Published:** 2020-07-13

**Authors:** Valeria Santini, Pierre Fenaux, Aristoteles Giagounidis, Uwe Platzbecker, Alan F. List, Torsten Haferlach, Jim Zhong, Chengqing Wu, Konstantinos Mavrommatis, C. L. Beach, Kyle J. MacBeth, Antonio Almeida

**Affiliations:** 1 MDS UNIT, Hematology, Azienda Ospedaliero Universitaria Careggi, University of Florence, Florence, Italy; 2 Service d’Hématologie Séniors, Hôpital Saint-Louis, Université Paris 7, Paris, France; 3 Department of Oncology, Hematology, and Palliative Care, Marien Hospital Düsseldorf, Düsseldorf, Germany; 4 Medical Clinic and Policlinic I, Hematology and Cellular Therapy, Leipzig University Hospital, Leipzig, Germany; 5 Formerly Department of Malignant Hematology, H. Lee Moffitt Cancer Center and Research Institute, Tampa, FL USA; 6 MLL Munich Leukemia Laboratory, Munich, Germany; 7 Bristol Myers Squibb, Princeton, NJ, USA; 8 Instituto Português de Oncologia de Lisboa Francisco Gentil, Lisbon, Portugal

**Keywords:** Myelodysplastic syndrome, Cancer genetics

## To the Editor

Myelodysplastic syndromes (MDS) are bone marrow neoplasias that involve mutations in genes affecting various cell pathways [1]. Both clonal evolution and the type of somatic mutations appear to be associated with response to treatment [1, 2]. Response to hypomethylating agents possibly correlates with mutations in *TET2* [3], *TP53* [4], and mutation burden [5]. Erythroid response to erythropoiesis-stimulating agents (ESAs) has also been linked to mutation burden [6]. Recently, luspatercept has been shown to enhance late-stage erythropoiesis in non-del(5q) MDS with ring sideroblasts regardless of mutational status [7].

Lenalidomide therapy has previously been investigated in MDS-005 (NCT01029262); a randomized, phase 3 trial of ESA-refractory or -ineligible patients with lower-risk non-del(5q) MDS [8]. Previous studies investigating markers of response to lenalidomide, alone or in combination with ESAs, lacked statistical power and require independent validation [9, 10]. We carried out an exploratory investigation into the relationship between gene mutations and response to lenalidomide in patients from the MDS-005 study.

Efficacy endpoints from the MDS-005 trial were analyzed according to gene mutation status [11]. Gene expression analysis was performed at baseline as a primary endpoint of the MDS-005 study, other analyses were exploratory endpoints. DNA was isolated from bone marrow mononuclear cells (*n* = 177) or whole blood (*n* = 21) collected at screening from consenting patients with adequate tissue for exploratory biomarker analysis. Targeted next-generation sequencing of 56 genes was performed at the Munich Leukemia Laboratory (Supplementary Table 1).

Baseline characteristics of the biomarker cohort (*N* = 198; 130/68 received lenalidomide/placebo) were generally comparable to those of patients not included (Supplementary Fig. 1; Supplementary Table 2). Within the biomarker cohort, baseline characteristics were well balanced between patients receiving lenalidomide and those receiving placebo (Supplementary Table 3). Baseline characteristics of biomarker cohort patients by *ASXL1* mutation status are shown in Supplementary Table 4. In total, 173 (87.4%) harbored at least 1 mutation in the 56 genes assessed (Supplementary Table 5). Fifty-five patients (27.8%) had a single mutation, 63 (31.8%) had 2 mutations, and 55 (27.8%) had >2 mutations. Mutations were detected in 30 of the 56 genes assessed (Supplementary Fig. 2a). The most commonly observed gene mutations were *SF3B1* (58.6% of patients)*, TET2* (33.0%)*, ASXL1* (23.2%), and *DNMT3A* (13.6%). The most frequently observed co-mutations were *SF3B1* and *TET2* (22.7%), *SF3B1* and *DNMT3A* (10.1%), *SF3B1* and *ASXL1* (9.6%), and *TET2* and *ASXL1* (8.6%) (Supplementary Fig. 2b); 99.1% of patients with *SF3B1* mutations had ring sideroblasts ≥5% (Supplementary Table 6). The relationship between mutation status and cytogenetic abnormalities at baseline is shown in Supplementary Table 7.

**Fig. 1:**
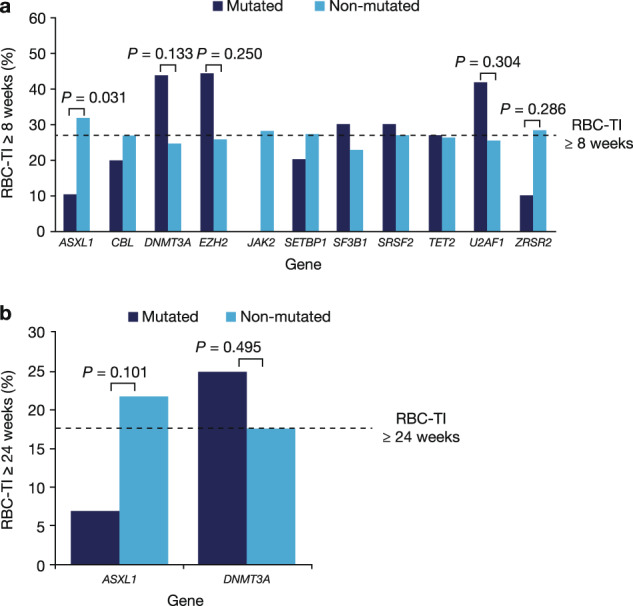
Proportion of patients treated with lenalidomide who achieved RBC-TI according to gene mutation status. RBC-TI ≥8 weeks (**a**) and ≥24 weeks (**b**). Dotted lines represent response rates in all patients treated with lenalidomide (26.9% and 17.5% for RBC-TI ≥8 and ≥24 weeks, respectively). RBC-TI red blood cell transfusion independence.

Among lenalidomide-treated patients, the proportion of patients achieving red blood cell transfusion independence (RBC-TI) ≥8 weeks was significantly lower in those with *ASXL1* mutations than those without (10.3% vs. 31.7%; *p* = 0.031) (Fig. 1a). The proportion of patients achieving RBC-TI ≥8 weeks was nominally higher in those with *DNMT3A* mutations than in those without (43.8% vs. 24.6%; *p* = 0.133). RBC-TI ≥8 weeks was achieved by 42.9% of patients with both *DNMT3A* and *SF3B1* mutations (which co-occurred in most patients), 1 of 2 patients (50.0%) with *DNMT3A* only, 27.1% of patients with *SF3B1* only, and 21.8% of patients with neither *DNMT3A* nor *SF3B1* mutations. Eleven patients (5.6%) had *EZH2* mutations; of the 9 receiving lenalidomide, 4 (44.4%) achieved RBC-TI ≥8 weeks, versus 31 of 121 (25.6%) patients without *EZH2* mutations (*p* = 0.250) (Fig. 1a). Mutations in other genes were not significantly associated with response to lenalidomide.

No specific single mutation was associated with achievement of RBC-TI ≥24 weeks with lenalidomide, although patients with *ASXL1* mutations had a nominally lower rate of RBC-TI ≥24 weeks than those without *ASXL1* mutations (6.9% vs. 21.8%; *p* = 0.101) (Fig. 1b). Mutation status of the 12 patients who achieved RBC-TI ≥52 weeks is shown in Supplementary Table 8. There was no association between mutation status and duration of RBC-TI in patients treated with lenalidomide.

The number of mutations present did not appear to correlate with response, and no relationship was found between the variant allele frequency of mutations and response rates (Supplementary Fig. 3). Of 27 lenalidomide-treated patients evaluable for cytogenetic response, 5 (18.5%) achieved cytogenetic complete response and 4 (14.8%) had cytogenetic partial response (PR) (Mutations status: Supplementary Table 9). Presence of an A/G polymorphism at rs1672753 in the *CRBN* gene was not associated with response in the MDS-005 study population. No patients evaluated in our study had a *CSNK1A1* mutation.

**Fig. 2:**
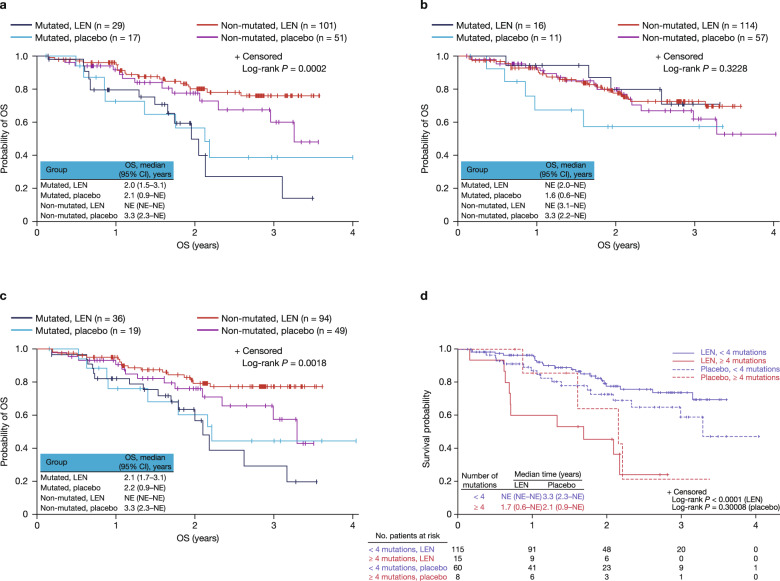
Overall survival by treatment group and mutation status. For *ASXL1* (**a**), *DNMT3A* (**b**), any 1 of 5 genes in the Bejar 5-gene mutation signature (**c**), and number of mutations (**d**). CI confidence interval, LEN lenalidomide, NE not estimable, OS overall survival.

Overall survival (OS) by treatment group and *ASXL1* mutation status is shown in Fig. 2a. Mutations in the *ASXL1* gene were associated with significantly shorter median OS, regardless of treatment group (log-rank *p* < 0.0001). Among patients with *ASXL1* mutations, median OS was 2.0 years (95% confidence interval (CI) 1.5–3.1) with lenalidomide and 2.1 years (95% CI 0.9–not estimable (NE)) with placebo (*p* = 0.576). Shorter OS in patients with *ASXL1* mutations was confirmed with a Cox proportional hazards model (hazard ratio (HR) 2.6 [95% CI 1.5–4.6]) controlling for clinical covariates including baseline erythropoietin (EPO), blast percentage, and Revised International Prognostic Scoring System (IPSS-R) risk. The presence of *DNMT3A* mutations had no significant effect on OS in patients receiving placebo (log-rank *p* = 0.3228) (Fig. 2b); however, there was a trend toward longer median OS with lenalidomide (NE [95% CI 2.0 years–NE]) compared with placebo (1.6 years [95% CI 0.6–NE]) (*p* = 0.123). Mutations in *SF3B1* and *TET2* had no significant effect on OS.

The presence of a mutation in any of 5 genes previously linked to poor prognosis (that is *ASXL1, ETV6, EZH2, RUNX1*, and *TP53*) [12] was associated with shorter median OS (log-rank *p* = 0.0018) (Fig. 2c). Median OS was 2.1 years (range 1.7–3.1) in lenalidomide-treated patients with mutations in any of the 5 genes versus NE (NE–NE) in non-mutated lenalidomide-treated patients (*p* = 0.0003).

In patients receiving lenalidomide, a higher number of mutations was significantly associated with shorter median OS (log-rank *p* = 0.0005) (Fig. 2d). Median OS was NE in patients with 0–3 mutations and 1.7 years (range 0.6–NE) in patients with ≥4 mutations.

Patients with ≥4 mutations were more likely to have a high serum EPO level (>500 mU/ml) than patients with fewer mutations (*p* = 0.0190). Int-1-risk patients with high EPO levels were more likely to have ≥4 mutations than those with low EPO levels (10/16 patients [62.5%] versus 6/16 patients [37.5%]; *p* = 0.0962); there was no significant difference for Low-risk patients (3/6 patients [50.0%] vs. 3/6 patients [50.0%]; *p* = 0.3403). Int-1-risk patients with high EPO levels were also more likely to have mutations in any of the 5 genes previously linked to poor prognosis (*ASXL1, ETV6, EZH2, RUNX1*, and *TP53*) [12] compared to those with low EPO levels (60.6% vs. 39.4%; *p* = 0.0102); there was no significant difference for Low-risk patients.

The high prevalence of mutations and mutational profile are consistent with results from a previous analysis of patients with MDS [1, 2, 12]. The effect of *ASXL1* mutations on response rate to lenalidomide and OS, regardless of treatment, is consistent with prior reports showing an adverse prognostic effect of *ASXL1* mutations [2, 12, 13]. Also, the trend towards improved response to lenalidomide in patients with *DNMT3A* mutations is consistent with results from a trial of patients with non-del(5q) MDS in which *DNMT3A* mutations predicted better response [10]. Patients with *DNMT3A* mutations have higher rates of progression to acute myeloid leukemia (AML) [14]. It may therefore be that the trend towards improved OS in *DNMT3A*-mutated patients receiving lenalidomide in this study was a result of a reduction in the rate of AML progression.

The association of increased mutation burden with worse OS is consistent with previous reports of a link with shorter leukemia-free survival [1]. Patients with ≥4 mutations were more likely to have a high serum EPO level (>500 mU/ml), a negative prognostic marker associated with a lower probability of response to treatment [8, 15]. High serum EPO level was associated with the presence of mutations in any of 5 poor-prognosis genes (*ASXL1, ETV6, EZH2, RUNX1*, and *TP53*) [13] and specifically *ASXL1*. Taken together with the observed association between *ASXL1* mutations and low response rate to lenalidomide, this provides further insight into previous observations that patients with high baseline EPO level have a lower probability of responding to lenalidomide [8].

Limitations of this analysis include the confounding effects of co-mutations on outcomes and the relatively small and heterogeneous group of patients.

A better understanding of how somatic mutations influence lenalidomide response may help identify patients with lower-risk non-del(5q) MDS who are most likely to respond to lenalidomide therapy. Efforts are currently ongoing to incorporate mutations into the IPSS-R to improve its prognostic value [13]. Currently, there are no unequivocal genetic markers of response to non-therapeutic targets, such as lenalidomide for lower-risk non-del(5q) MDS. However, our finding that *ASXL1* mutations negatively affect outcomes with lenalidomide is relevant, as negative prognostic factors are not always predictive of poor response, as was demonstrated by the contrasting finding for *TP53* in MDS patients treated with decitabine [4]. Thus, identifying predictive markers of response to lenalidomide in non-del(5q) MDS patients remains an ongoing challenge.

## Supplementary information

Supplementary Appendix
