# Supplementary material for: Impact of somatic mutations on response to lenalidomide in lower-risk non-del(5q) myelodysplastic syndromes patients
Source: Leukemia. 2020 Jul 13;35(3):897–900. doi: 10.1038/s41375-020-0961-3 (PMC7932918; doi:10.1038/s41375-020-0961-3)
Supplement: Supplementary file 1 — Supplementary Appendix [file 41375_2020_961_MOESM1_ESM.docx]

**Supplemental Data**

[Supplementary Materials and methods 2](#_Toc43996589)

[MDS-005 study design 2](#_Toc43996590)

[Mutational analysis 2](#_Toc43996591)

[Supplemental Table 1 Panel of 56 genes included in the study 3](#_Toc43996592)

[Statistical analyses 3](#_Toc43996593)

[Supplementary results 3](#_Toc43996594)

[AML progression 3](#_Toc43996595)

[Supplemental Table 2 Baseline characteristics of patients included and not included in the biomarker cohort study (*N* = 239) 4](#_Toc43996596)

[Supplemental Table 3 Baseline characteristics of patients in the biomarker cohort (N = 198) 7](#_Toc43996597)

[Supplemental Table 4 Baseline characteristics of patients receiving lenalidomide in the biomarker cohort study by *ASXL1* mutation status (*N* = 130) 8](#_Toc43996598)

[Supplemental Table 5 Number of mutated genes per patient 10](#_Toc43996599)

[Supplemental Table 6 Relationship between *SF3B1* mutation status and presence of ring sideroblasts 11](#_Toc43996600)

[Supplemental Table 7 Mutation status and cytogenetic abnormalities at baseline 12](#_Toc43996601)

[Supplemental Table 8 Mutation status of the 12 patients treated with lenalidomide who achieved RBC-TI ≥ 52 weeks 14](#_Toc43996602)

[Supplemental Table 9 Mutation status of the 9 patients treated with lenalidomide who achieved cytogenetic response 15](#_Toc43996603)

[Supplemental Fig. 2 Gene mutations. Frequency of gene mutations (a) and co-mutations (b). 17](#_Toc43996604)

[Supplemental Fig. 3 VAF of mutations and RBC-TI ≥ 8 weeks response in lenalidomide-treated patients 19](#_Toc43996605)

# Supplementary Materials and methods

## MDS-005 study design

The MDS-005 study was approved by the institutional review boards of participating centers and conducted according to the Declaration of Helsinki. All patients in the MDS-005 trial provided written consent.

## Mutational analysis

The next-generation sequencing libraries were generated with the ThunderBolts™ myeloid panel (RainDance Technologies) and sequenced on an Illumina MiSeq system (San Diego, CA, USA) with a mean coverage of 2341× and a minimum coverage of 100× for both reads (Read 1 and Read 2), reaching a sensitivity of 3%. Raw reads were processed with JSI Sequence Pilot software (v4.1.1 Build 510, JSI medical systems, Kippenheim, Germany) for alignment (hg19) and somatic variant calling. Protein truncating and frameshift variants were classified as mutations. Non-synonymous changes were included if they were well annotated (several definite submissions to COSMIC v74, IARC r17 [in case of TP53], or ClinVar). Other non-protein truncating variants were defined as variants of uncertain significance (VUS) or polymorphisms depending on their presence in dbSNP (build 144) and in-silico functional impact computed by PolyPhen-2 and SIFT (v1.03). Single nucleotide polymorphism rs1672753 was genotyped in DNA extracted from bone marrow mononuclear cells using an Applied Biosystems TaqMan assay (Thermo Fisher Scientific, Waltham, MA, USA).

## Supplemental Table 1 Panel of 56 genes included in the study

| *ASXL1* | *CRBN* | *FLT3TKD* | *JAK1* | *MPL* | *PTPN11* | *STAG2* |
| --- | --- | --- | --- | --- | --- | --- |
| *BCOR* | *CSF1R* | *GATA1* | *JAK2* | *MYD88* | *RAD21* | *STAT3* |
| *BCORL1* | *CSF3R* | *GATA2* | *JAK3* | *NOTCH1* | *RUNX1* | *TET2* |
| *BRAF* | *CSNK1A1* | *GNAS* | *KDM6A* | *NPM1* | *SETBP1* | *TP53* |
| *CALR* | *DDX41* | *GNB1* | *KIT* | *NRAS* | *SF3B1* | *U2AF1* |
| *CBL* | *DNMT3A* | *HRAS* | *KRAS* | *PHF6* | *SMC1A* | *U2AF2* |
| *CBLB* | *ETV6* | *IDH1* | *MAP2K1* | *PML* | *SMC3* | *WT1* |
| *CEBPA* | *EZH2* | *IDH2* | *MLL* | *PTEN* | *SRSF2* | *ZRSR2* |

## Statistical analyses

The Fisher exact test was used to test the association between mutation status and response. Overall survival was characterized using the Kaplan–Meier method, with differences evaluated by the log-rank test. The data cutoff for inclusion in this analysis was March 17, 2014, unless otherwise specified.

# Supplementary results

## AML progression

The incidence of AML progression was 1.91 (95% CI, 0.80 to 4.59) per 100 person-years among patients receiving lenalidomide and 2.46 (95% CI, 0.79 to 7.64) per 100 person-years among patients receiving placebo.

## Supplemental Table 2 Baseline characteristics of patients included and not included in the biomarker cohort study (*N* = 239)

| **Characteristic** | **Included (*n* = 198)** | **Not included (*n* = 41)** |
| --- | --- | --- |
| **Age, median (range), years** | 71 (43–87) | 70 (53–85) |
| Age ≥ 65 years, *n* (%) | 145 (73.2) | 27 (65.9) |
| Male, *n* (%) | 132 (66.7) | 30 (73.2) |
| **Region, *n* (%)** |  |  |
| North American/Australia | 18 (9.1) | 7 (17.1) |
| Europe | 169 (85.4) | 33 (80.5) |
| Japan | 11 (5.6) | 1 (3.4) |
| **ECOG performance status, *n* (%)** |  |  |
| 0 | 102 (51.5) | 19 (46.3) |
| 1 | 87 (43.9) | 20 (48.8) |
| 2 | 9 (4.5) | 2 (4.9) |
| Time since diagnosis, median (range), years | 2.8 (0.1–29.6) | 2.4 (0.4–1.67) |
| **WHO 2008 category (central review), *n* (%)** |  |  |
| RA | 1 (0.5) | 1 (2.4) |
| RCUD | 4 (2.0) | 1 (2.4) |
| RARS | 15 (7.6) | 4 (9.8) |
| RCMD | 146 (73.7) | 28 (68.3) |
| RAEB-1 | 32 (16.2) | 7 (17.1) |
| **IPSS risk, *n* (%)** |  |  |
| Low | 96 (48.5) | 19 (46.3) |
| Int-1 | 102 (51.5) | 22 (53.7) |
| **Karyotype IPSS (central review), *n* (%)** |  |  |
| Good | 166 (83.8) | 31 (75.6) |
| Intermediate | 31 (15.7) | 10 (24.4) |
| Poor | 0 (0) | 0 (0) |
| Missing | 1 (0.5) | 0 (0) |
| **Number of cytopenias, *n* (%)** |  |  |
| 0 | 12 (6.1) | 4 (9.8) |
| 1 | 105 (53.0) | 21 (51.2) |
| 2 | 73 (36.9) | 15 (36.6) |
| 3 | 7 (3.5) | 1 (2.4) |
| Missing | 1 (0.5) | 0 (0) |
| **Serum erythropoietin, *n* (%), mU/ml** |  |  |
| ≤500 | 124 (62.6) | 23 (56.1) |
| >500 | 69 (34.8) | 17 (41.5) |
| Missing | 5 (2.5) | 1 (2.4) |
| pRBC transfusion burden, median (range), units/28 days | 3.0 (1.5–8.8) | 3.5 (1.8–9.8) |
| **Prior therapy for MDS, *n* (%)** | 166 (83.8) | 34 (82.9) |
| Prior ESA | 156 (78.8) | 32 (78.0) |
| Prior G-CSF | 31 (15.7) | 7 (17.1) |

ECOG, Eastern Cooperative Oncology Group; ESA, erythropoiesis-stimulating agent; G-CSF, granulocyte colony-stimulating factor; IPSS, International Prognostic Scoring System; pRBC, packed red blood cell; RA, refractory anemia; RAEB, RA with excess blasts; RARS, RA with ring sideroblasts; RCMD, refractory cytopenia with multilineage dysplasia; RCUD, refractory cytopenia with unilineage dysplasia; WHO, World Health Organization.

## Supplemental Table 3 Baseline characteristics of patients in the biomarker cohort (N = 198)

| **Characteristic** | **Lenalidomide (*n* = 130)** | **Placebo (*n* = 68)** |
| --- | --- | --- |
| Age, median (range), years | 71.0 (46.0–87.0) | 70.5 (43.0–87.0) |
| Male, *n* (%) | 86 (66.2) | 46 (67.6) |
| Time since diagnosis, median (range), years | 3.0 (0.1–29.6) | 2.6 (0.3–20.2) |
| pRBC transfusion burden, median (range), units/28 days | 3.0 (1.8–8.8) | 3.3 (1.5–7.3) |
| **IPSS risk, *n* (%)** |  |  |
| Low | 67 (51.5) | 29 (42.6) |
| Int-1 | 63 (48.5) | 39 (57.4) |
| **WHO 2008 category (central review), *n* (%)** |  |  |
| RA | 1 (0.8) | 0 (0) |
| RCUD | 4 (3.1) | 0 (0) |
| RARS | 8 (6.2) | 7 (10.3) |
| RCMD | 94 (72.3) | 52 (76.5) |
| RAEB-1 | 23 (17.7) | 9 (13.2) |

IPSS, International Prognostic Scoring System; pRBC, packed red blood cell; RA, refractory anemia; RAEB, RA with excess blasts; RARS, RA with ring sideroblasts; RCMD, refractory cytopenia with multilineage dysplasia; RCUD, refractory cytopenia with unilineage dysplasia; WHO, World Health Organization.

## Supplemental Table 4 Baseline characteristics of patients receiving lenalidomide in the biomarker cohort study by *ASXL1* mutation status (*N* = 130)

| **Characteristic** | ***ASXL1*-mutated (*n* = 29)** | **ASXL1 non-mutated (*n* = 101)** |
| --- | --- | --- |
| Time since diagnosis, median (range), years | 1.1 (0.2 –13.9) | 3.5 (0.1–29.6) |
| **WHO 2008 category (central review), *n* (%)** |  |  |
| RA | 1 (3.4) | ­­0 |
| RCUD | 1 (3.4) | 3 (3.0) |
| RARS | 0 | 8 (7.9) |
| RCMD | 21 (72.4) | 73 (72.3) |
| RAEB-1 | 6 (20.7) | 17 (16.8) |
| **IPSS risk (investigator), *n* (%)** |  |  |
| Low | 15 (51.7) | 52 (51.5) |
| Int-1 | 14 (48.3) | 49 (48.5) |
| **Karyotype IPSS (central review), *n* (%)** |  |  |
| Good | 26 (89.7) | 84 (83.2) |
| Intermediate | 3 (10.3) | 16 (15.8) |
| Poor | 0 | 0 |
| Missing | 0 | 1 (1.0) |
| **Number of cytopenias, *n* (%)** |  |  |
| 0 | 2 (6.9) | 5 (5.0) |
| 1 | 15 (51.7) | 56 (55.4) |
| 2 | 10 (34.5) | 38 (37.6) |
| 3 | 2 (6.9) | 2 (2.0) |
| Missing |  |  |
| **Serum erythropoietin, *n* (%), mU/ml** |  |  |
| ≤500 | 13 (44.8) | 69 (68.3) |
| >500 | 15 (51.7) | 29 (28.7) |
| Missing | 1 (3.4) | 3 (3.0) |
| pRBC transfusion burden, median (range), units/28 days | 3.0 (2.0–8.8) | 3.0 (1.8–6.8) |

IPSS, International Prognostic Scoring System; pRBC, packed red blood cell; RA, refractory anemia; RAEB, RA with excess blasts; RARS, RA with ring sideroblasts; RCMD, refractory cytopenia with multilineage dysplasia; RCUD, refractory cytopenia with unilineage dysplasia; WHO, World Health Organization.

## Supplemental Table 5 Number of mutated genes per patient

| **Mutation status** | **Number of patients (%) (*N* = 198)** | **Lenalidomide response rate**, n/N (%) |
| --- | --- | --- |
| **At least 1 mutation** | 173 (87.4) | - |
| 1 mutation | 55 (27.8) | 12/36 (33.3) |
| 2 mutations | 63 (31.8) | 10/44 (22.7) |
| 3 mutations | 32 (16.2) | 5/20 (25.0) |
| 4 mutations | 17 (8.6) | 3/12 (25.0) |
| 5 mutations | 5 (2.5) | 1/3 (33.3) |
| 6 mutations | 1 (0.5) | - |
| No mutations | 25 (12.6) | 4/15 (26.7) |

^a^ 56-day RBC-TI response rate

## Supplemental Table 6 Relationship between *SF3B1* mutation status and presence of ring sideroblasts

| **Ring sideroblast status, %** | ***SF3B1* mutation status, *n* (%)** | |
| --- | --- | --- |
|  | **Mutated (*n* = 116)** | **Non-mutated (*n* = 82)** |
| **WHO 2008 criteria^a^** |  |  |
| <15 | 2 (1.7) | 54 (65.9) |
| ≥15 | 114 (98.3) | 28 (34.1) |
| **WHO 2016 criteria^b^** |  |  |
| <5 | 1 (0.9) | 46 (56.1) |
| ≥5 | 115 (99.1) | 36 (43.9) |

^a^ Vardiman JW, Thiele J, Arber DA, Brunning RD, Borowitz MJ, Porwit A *et al*. The 2008 revision of the World Health Organization (WHO) classification of myeloid neoplasms and acute leukemia: rationale and important changes. *Blood* 2009; **114**: 937-951.
^b^ Arber DA, Orazi A, Hasserjian R, Thiele J, Borowitz MJ, Le Beau MM *et al*. The 2016 revision to the World Health Organization classification of myeloid neoplasms and acute leukemia. *Blood* 2016; **127**: 2391-2405.

## Supplemental Table 7 Mutation status and cytogenetic abnormalities at baseline

| **Cytogenetic abnormality** | **Patients, *n*** | **Patients with mutations, *n*** | **Mutation type (patients, *n*)** |
| --- | --- | --- | --- |
| +8 | 20 | 19 | *SF3B1* (17) *DNMT3A* (5) *TET2* (4) *ASXL1* (3) *JAK2* (3) *STAG2* (2) *EZH2* (1) *IDH1* (1) *SRSF2* (1) *ZRSR2* (1) |
| -Y | 10 | 9 | *SF3B1* (5) *TET2* (4) *ASXL1* (3) *ZRSR2* (3) *EZH2* (1) *RUNX1* (1) *STAG2* (1) |
| 20q- | 4 | 2 | *ASXL1* (1) *CBL* (1) *SF3B1* (1) |
| 17p- | 1 | 1 | *ASXL1* (1) *SETBP1* (1) *SRSF2* (1) |
| Balanced translocation | 1 | 1 | *ASXL1* (1) *SF3B1* (1) *U2AF1* (1) |
| Other abnormalities | 14 | 13 | *ASXL1* (6) *SF3B1* (5) *JAK2* (2) *SETBP1* (2) *SRSF2* (2) *BCOR* (1) *CSF3R* (1) *ETV6* (1) *EZH2* (1) *TET2* (1) *TP53* (1) *U2AF1* (1) |

Patients may have had > 1 abnormality or mutation. None of the patients had a complex karyotype (3 or more cytogenetic abnormalities), and the following abnormalities were not observed: -5, 5q-, -7, 7q-, 12p-, t(11q23), 3q21, and 3q26.

## Supplemental Table 8 Mutation status of the 12 patients treated with lenalidomide who achieved RBC-TI ≥ 52 weeks

| **Patient number** | **Duration of RBC-TI, days** | **Mutated genes** |
| --- | --- | --- |
| 1 | 402 | *DNMT3A*, *EZH2*, *SF3B1* |
| 2 | 414 | *SF3B1*, *TET2* |
| 3 | 454 | *SF3B1* |
| 4 | 498 | *SF3B1* |
| 5 | 530 | *SF3B1*, *TET2* |
| 6 | 534 | *U2AF1* |
| 7 | 700 | Not evaluated |
| 8 | 709 | *SRSF2* |
| 9 | 1 133 | *SF3B1*, *TET2* |
| 10 | 1 295 | None |
| 11 | 1 332 | None |
| 12 | 1 396 | *SF3B1* |

RBC-TI, red blood cell transfusion independence.

## Supplemental Table 9 Mutation status of the 9 patients treated with lenalidomide who achieved cytogenetic response

| **Patient number** | **CyR** | **Mutated genes** |
| --- | --- | --- |
| 1 | Complete CyR | *SF3B1* |
| 2 | Complete CyR | *SF3B1* |
| 3 | Complete CyR | *SF3B1, TET2* |
| 4 | Complete CyR | *SF3B1, DNMT3A* |
| 5 | Complete CyR | *SF3B1, JAK2, STAG2* |
| 6 | Partial CyR | *JAK2* |
| 7 | Partial CyR | *SF3B1* |
| 8 | Partial CyR | *SF3B1* |
| 9 | Partial CyR | *SF3B1, DNMT3A, EZH2* |

CyR, cytogenetic response. Supplemental Fig. 1 Patient disposition of the biomarker study cohort


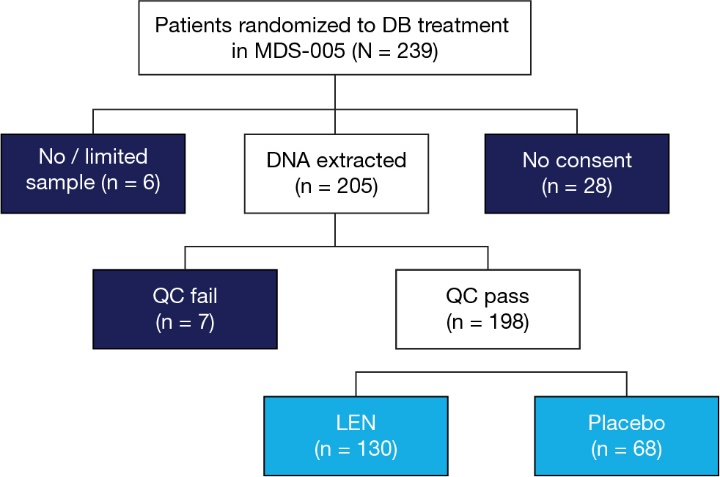


DB, double blind; LEN, lenalidomide; QC, quality control.

Supplemental Fig. 2 Gene mutations. Frequency of gene mutations (a) and co-mutations (b). Genes with no mutations detected: *BCORL1, CALR, CBLB, CEBPA, CRBN, CSF1R, CSNK1A1, FLT3TKD, GATA1, GATA2, GNB1, HRAS, JAK1, JAK3, KIT, MAP2K1, MLL, NOTCH1, NPM1, PML, PTEN, PTPN11, SMC1A, SMC3, U2AF2, WT1*

**a**
**
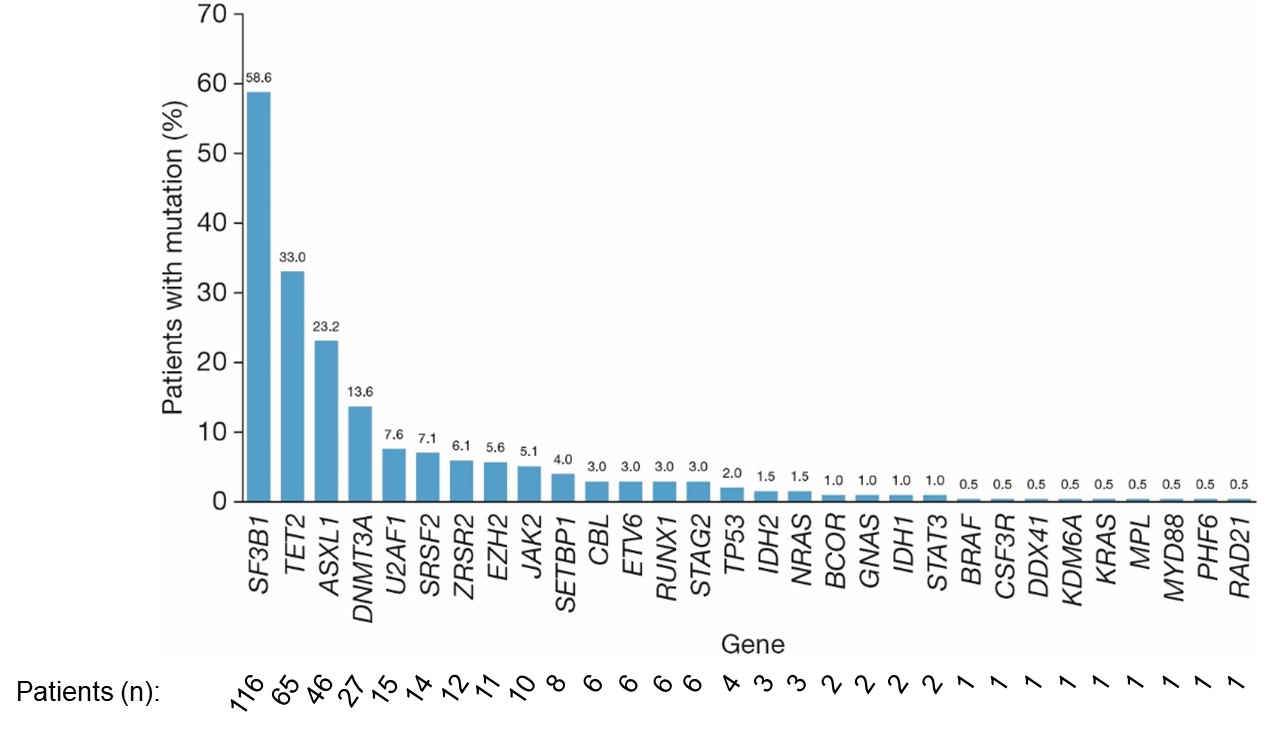
**

**b**

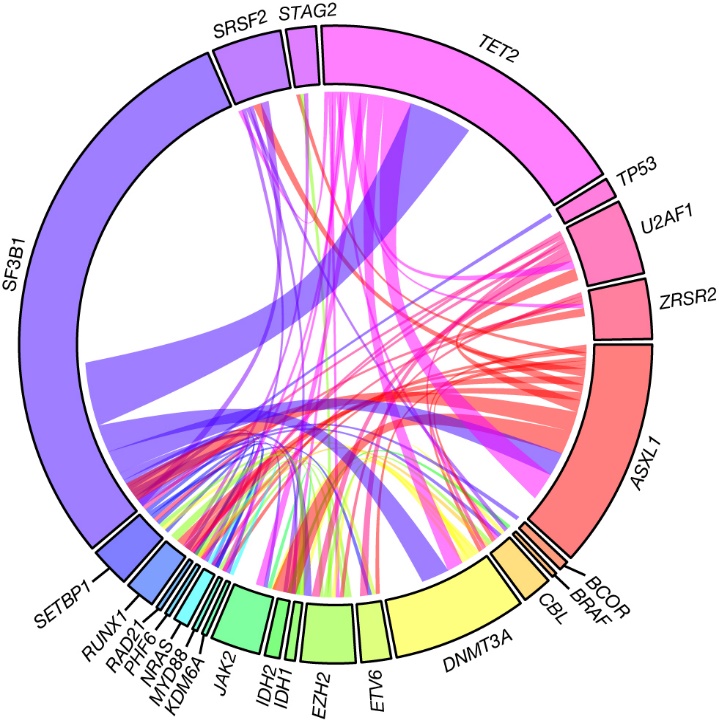


## Supplemental Fig. 3 VAF of mutations and RBC-TI ≥ 8 weeks response in lenalidomide-treated patients


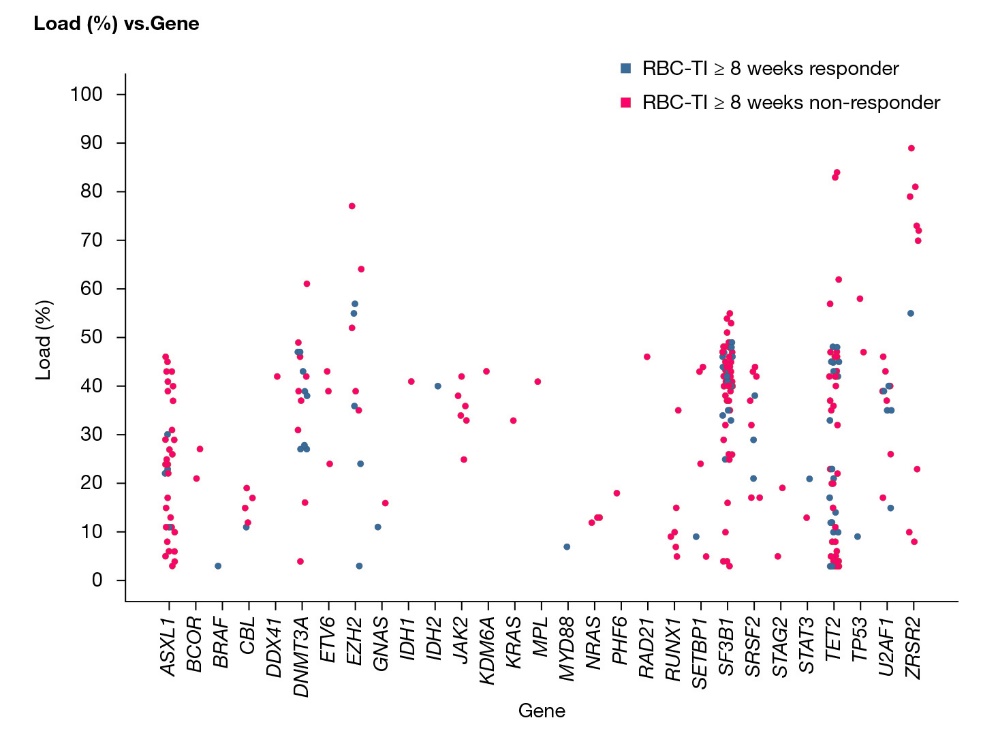


VAF, variant allele frequency.
